# Well prepared yet uncertain: Experiences of the early career transition after affiliation with an interdisciplinary graduate school

**DOI:** 10.1371/journal.pone.0321039

**Published:** 2025-04-29

**Authors:** Catharina Melander, Charlotte Löfqvist, Maria Haak, Søren E. Smedegaard Bengtsen, Gudrun Edgren, Susanne Iwarsson

**Affiliations:** 1 Department of Health, Education and Technology, Division of Nursing and Biomedical Engineering, Luleå University of Technology, Luleå, Sweden; 2 Department of Health Sciences, Faculty of Medicine, Lund University, Lund, Sweden; 3 Department of Nursing Education and Integrated Health Sciences, Faculty of Health Science, Kristianstad University, Kristianstad, Sweden; 4 Department of Educational Philosophy and General Education, Danish School of Education, Aarhus University, Aarhus, Denmark; 5 Centre for Teaching and Learning, Faculty of Medicine, Lund University, Lund, Sweden; Federal University of the ABC: Universidade Federal do ABC, BRAZIL

## Abstract

To contribute to the development of effective support for early career researchers during and after doctoral education, the aim of this study was to examine the experiences of the early career transition after affiliation with an interdisciplinary graduate school, using the Swedish National Graduate School on Ageing and Health (SWEAH) as an example. Through qualitative interviews with 18 alumni, the findings reveal that the graduate school had fostered interdisciplinary research skills among early career researchers, and they felt well-prepared for the next career stage. However, they expressed a need for continuous career support and personal guidance while still feeling confident and open-minded. Interdisciplinary graduate schools can play a crucial role in nurturing the next generation of early career researchers, providing them with the skills and identities needed for impactful research and collaboration in a complex, interconnected world. We suggest that academic careers should be understood in an entangled or interlinked way from the very beginning of the academic journey, and introduce a new conceptual coinage of “career ecologies”. This implies that doctoral students and early career researchers should be encouraged, empowered, energized, and invited to activate and make use of their societal voice, influence, and value, right from the early formative doctoral years. Moreover, strategic support is vital to maintain an interdisciplinary spirit and facilitate informed career choices.

## Introduction

Being an early career researcher implies diverse challenges, and has been described as a time permeated with insecurity and stress, partly due to a lack of support and precarious employment situations [1]. Maintaining realistic career expectations, preparing for diverse career paths, and adopting strategies to navigate these challenges effectively have been highlighted [2]. While such navigation is a skill that should be developed by the individual researcher, it is the responsibility of higher education institutions to support academic career development. Aspiring to contribute to the development of effective support for early career researchers during and after doctoral education, this paper focuses on the experiences of alumni who had graduated from an interdisciplinary graduate school which explicitly aimed to support the academic career of future research leaders.

The early career as a researcher begins after graduating from doctoral education. Doctoral education has gone through several changes in recent decades, such as improved supervision, better conditions for students, an increased interest in transferable skills, and quality assurance [3–7], resulting in a growing formalization, and growth in the number of graduate schools [3,4]. Still, early career researchers encounter challenges in career transitions, such as bridging the gap between their specialized research skills and the broader competencies required in diverse professional environments [cf. 8,9]. Doctoral education is integrated with ongoing research, where increased competition for funding and a growing focus on societal challenges have led to the development of interdisciplinary team science [cf. 10]. Interdisciplinary approaches to research are common in the field of ageing and health, which is the main interest of the present paper.

As problem-oriented interdisciplinary approaches are the foundation for many research fields, preparing early career researchers to synthesize information and knowledge from diverse disciplines and knowledge sources into coherent wholes is important [cf. 11]. Interdisciplinary research requires the complex and close connections between epistemic, institutional, social, and ideological perspectives to be addressed and taken into considerations [12,13], and there is often a negotiation of identities. For example, because conceptual and methodological models for interdisciplinary research are often lacking, challenges include collaborating with other researchers to establish a common epistemic foundation [e.g., 11,14,15].

Studies striving to deepen the understanding of former doctoral students’ perceptions of their early career experiences are scarce. Studies addressing the career transition from doctoral student to early career researcher are often framed from the perspectives of completion outcomes, supervision, and peer support [16]. There is a paucity of knowledge regarding doctoral students’ exposure to interdisciplinary perspectives during their education, which has been described as an iterative and reflexive journey and accomplishing a shared mindset with colleagues [17,18]. Interdisciplinary career paths are increasingly called for, in which complex problem-oriented societal challenges are addressed [cf. 13] and diverse methodological approaches and epistemological perspectives are needed [17]. Preparing for a research career in line with the needs of the 21^st^ century is not without its challenges [eg. [19]]. Interdisciplinary environments have been related to feelings such as a lack of cohesion, a lack of orientation, and unclear expectations [cf. 20,21], and could generate unintended conflicts that may hinder students’ progress [22].

Turning to opportunities, affiliation with an interdisciplinary graduate school can provide doctoral students with an academic connection to a specialized training program focused on a particular research field. Interdisciplinary graduate schools can be organized in various ways. For example, all doctoral students may be admitted to the same university, or the graduate school may function as a complement to the graduate education provided at the students’ home universities. Such complementary structures provide access to broader resources and networks across institutions, potentially enhancing students’ academic and professional development. Overall, the impact of being affiliated with an interdisciplinary graduate school during doctoral education on the early stages of a postdoctoral research career remains unclear. This study focused on the initial career progression of alumni of an interdisciplinary graduate school focusing on ageing and health, organized as a partnership between 13 universities.

Early career researchers have been defined in diverse ways, but most definitions include the first five years after the doctoral degree [23]. Conventional doctoral programs may not adequately prepare students for the diverse roles they must take on as early career researchers [24], and the transition from doctoral student to early career researcher might bring feelings of uncertainties related to career development. Such uncertainties are related to doubts about being able to develop original ideas and rigorous science, and to secure satisfying positions within or outside academia [2,25,26]. The early career phase includes developmental stages such as becoming confident, being recognized as a researcher, and becoming more productive and sophisticated in research [27,28], all of which can be influenced by the characteristics of a research field, such as its impact on the likelihood of pursuing geographic mobility [29]. While doctoral education aims to prepare graduates for employment in the wider labor market, many embark on doctoral programs with an interest in a university career, although securing a permanent job in academia is often challenging [cf. 2,25,26]. Hence, early career researchers may work in the public (governmental), private (business/industry), non-profit, or higher education sectors, with career paths varying by country, labor sector and discipline [16].

Moreover, interdisciplinary approaches imply additional challenges when it comes to career development. The academic labor market typically operates in disciplinary terms, and early career researchers need to provide outputs that can be recognized by future employers representing traditional academic disciplines [13]. Successful interdisciplinary careers require flexible structures that consider the unique challenges and benefits of interdisciplinary work [30]. Still, while institutions may promote interdisciplinary education, this support rarely extends to practice. Organizational structures, especially in academia, can still favor traditional, discipline-specific work, making it harder for interdisciplinary scholars to find spaces in which to apply their skills [cf. 28,31]. Studies exploring perceptions of early career experiences among students who focused on interdisciplinarity during their doctoral education are scarce. To strengthen the preparation for interdisciplinary research, there is a need to explore how such career preparation is perceived by early career researchers and how insights and experiences influence their career transition and their development as researchers. Accordingly, the aim of this study was to examine the experiences of the transition to early career researcher after affiliation with an interdisciplinary graduate school.

## Theoretical underpinnings

We applied an ecological approach, building on Ronald Barnett’s theory of the ecological university and ecological learning in higher education [32,33]. Barnett argues that in the 21st century, universities and higher education are inextricably interlinked with the wider societal contexts in which they are entangled and nested. Thus, it is important to understand universities not as separate, removed, or distanced from the wider societal (professional, political, cultural) contexts. Instead, universities continuously move within their ecological fields and establish new ecological configurations [32]. The generation of knowledge about how career transitions become both meaningful and possible depends on the understanding of a university’s institutional and societal spaces for agency, which Barnett [32, p. 27] referred to as “ecological agency”.

With Barnett’s theory, we get to understand early career researchers not as separate, individual entities moving from one realm (university) into another (societal, professional), but that they are already ecologically interlinked through their doctoral education and subsequent academic positions. Envisioning universities and higher education as an interconnected system that dynamically interacts with multiple ecosystems – epistemic, institutional, social, and ideological – makes this theoretical reasoning highly relevant to studying interdisciplinary career transitions for doctoral students. Barnett argues that to further qualify the university–society interconnections, while also being situated mainly within the university realm, we need to see how “the learner is also a learning ecology at a further level; namely, that of the interlocking of her/his own learning ventures with the learning processes of the wider world” [32, p. 108]. Thus, we move away from understanding learning as an isolated and individual trajectory. Instead, we integrate into our higher and doctoral education pedagogies the notion that students, and later early career researchers, should not be cast in the role of life-long learners, but as “lifewide learners”. These learners are “engaged in learning across multiple learning sites and seeking to make a continuing and integrated life-story out of those manifold experiences” [32, p. 111]. Ecological learning can be viewed as requiring reflexivity, adaptability, and negotiation between different knowledge systems, which is consistent with the challenges faced by interdisciplinary early career researchers in bridging disciplinary gaps and integrating broader competencies into different professional settings. If ecological agency is not developed and guided until postdoctoral level, it will be more difficult to enable successful career transitions than if an ecological pedagogy had been applied earlier in the process. By situating interdisciplinary research and career development within the perspective of the ecological university, Barnett’s theory highlights the need for flexible academic structures that support identity negotiation, epistemic foundations for collaboration, and resilience in navigating the labor market.

In addition, we drew inspiration from Inouye and Bengtsen [34], who built on Barnett’s theory of ecological agency in relation to academic career transitions. They point out that to develop an ecological curriculum in doctoral education, the ecological dimensions have to be integrated into academic practices such as genres of writing, research practices and methods, and supervision pedagogies, but also wider researcher horizons including “notions of citizenship and social justice” [34, p. 219]. To enable successful academic career transitions, the doctoral and postdoctoral researcher must be enabled pedagogically to understand herself as a key stakeholder not only in the university but also in society, who “may influence the surrounding societal contexts through her research, whether it be via structural external partnerships, research collaborations, or researching into a specific public or private institutions, civil initiatives, professional practices, social or cultural histories, and value systems” [34, p. 220].

Building further on Barnett’s ecological theory, Bengtsen [35] coined the concept of doctoral ecologies to include the pedagogical, curricular, and institutional preparation and support of doctoral and postdoctoral researchers long-term academic trajectories. Bengtsen [35, p. 151] underlines that it is central to encourage and sustain the understanding as early as from doctorate level that “the ecological power lies in the embeddedness and linkage itself” between academic and surrounding societal contexts. Pedagogy and mentoring alone cannot make this development successful; it will also depend “on forms of graduate school leadership that ensure synergy and links between formal, informal, and nonformal learning domains” [35, p. 157]. This, together with Barnett’s theory of the ecological university, provides a conceptual basis for understanding the tensions and opportunities inherent in interdisciplinary careers and outlines a vision to foster cohesion, guidance, and clear expectations throughout doctoral education and into the transition to an early career researcher. In this way, fostering and sustaining ecological learning and agency among early career researchers hinges on a more profound and thorough re-thinking and re-enacting of the doctoral and postdoctoral curriculum and connected forms of institutional leadership. By emphasizing iterative and reflexive learning, this approach offers a pathway to more sustainable and inclusive career paths, countering the disciplinary rigidity of traditional academic organizations.

## Methods

### Study design and approach

This study takes an explorative and inductive approach to gain an empirical understanding of a phenomenon where existing theories and research are limited. This involved cautiously exploring the topic in a flexible and open manner to discover new insights and patterns that may not yet be fully understood or documented [36]. We conducted qualitative semi-structured interviews with 18 alumni from an interdisciplinary graduate school, allowing us to gain an understanding of the participants’ subjective realities.

### Study context

Established in 2014, the Swedish National Graduate School on Ageing and Health (SWEAH) is an interdisciplinary learning environment with the overarching goal of promoting networking and ensuring access to a national base of researchers for future research on ageing and health [for details, see 18]. SWEAH annually announces a call for affiliation of doctoral students from 13 Swedish universities, with students admitted to doctoral education at their home universities while gaining access to networking opportunities, interdisciplinary courses, and learning activities as part of their affiliation, while also engaging supervisors and alumni. The choice of SWEAH as an example is driven by Sweden’s strong emphasis on supporting early career researchers, which makes it an appropriate landscape for studying career transitions. By fostering interdisciplinary research across diverse fields such as medicine and health, social sciences, and technology, SWEAH exemplifies the integration of broad academic perspectives. Thus, SWEAH serves as an ideal case for examining institutional factors influencing early career researchers in Sweden.

### Participants

We recruited participants who had been affiliated with SWEAH during doctoral education, with the inclusion criterion being that they had completed their PhD degree at least one year ago. We conducted recruitment in January 2021, inviting 22 alumni who met the inclusion criterion to participate. The first author (CM) approached them via e-mail with written information about the study and an invitation to participate. After presumptive participants indicated their interest, we contacted them by phone or via a video conferencing tool for additional information and an opportunity to answer their questions. Four declined due to health issues, heavy clinical workload, or childcare, leaving us with 18 early career researchers who gave their written consent to participate. These 18 participants had been affiliated with the graduate school during the first or second year of their doctoral studies, and continued their affiliation until graduation. They graduated during 2018–2020 and represented a variety of disciplines such as health and medical sciences, information and communication technology, packages logistics, psychology, and sociology. See Table 1 for details.

**Table 1:** Characteristics of the participants at the time of the interviews, N=18.

Characteristic	
N	18
Age (years)	30–60 (mean 39.8)
Gender	
Women (%)	83.3
Men (%)	16.7
Time since defense (months)	15–37 (mean 23.6)
Current employment	
Postdoctoral appointment (%)	66.7
Junior/senior lecturer (%)	16.7
Other (%)	16.7
Country of current employment (%)	
Sweden	72.2
Other (Australia, Europe, North America)	27.8

### Interview guide

We developed a semi-structured interview guide with open-ended questions influenced by the Researcher Development Framework [8], goals for doctoral education, and findings from a previous study focusing on doctoral students’ perceptions of being affiliated with the graduate school [18] (S1 Appendix). The questions addressed the following domains: Engagement, influence, and impact; Research governance, and organization and personal effectiveness; and Knowledge and intellectual abilities. The aim was to make participants describe and reflect upon their career transitions after graduation. We conducted a pilot interview to test the interview guide, which resulted in minor revisions to clarify some questions.

### Data collection

The authors CM and CL conducted individual semi-structured interviews during February and March 2021. To create a relaxed atmosphere where participants could talk freely, they strived to take on a stance of immediacy and presence during the interviews. Along with the interview guide, they asked probing and clarifying questions to gain rich descriptions. The interviews were audio recorded via a video conferencing tool, and each interview lasted 40–60 minutes. A professional transcription agency transcribed the interviews verbatim.

### Data analysis

We applied qualitative conventional content analysis [36] with an inductive approach to analyze textual data, where coding categories were derived directly from the data itself, without predefined theoretical frameworks. Treating the 18 interviews as one dataset, the transcribed interviews were entered into the NVivo software to share each step of interpretation between CM and CL. They held regular online meetings, e-mail correspondence, and telephone meetings during the iterative analysis process.

The analysis started with CM and CL reading the transcripts several times to get a sense of the whole and to become familiar with the data. They then extracted meaning units, grouped them together based on content similarities, and coded them accordingly. CM and CL continuously discussed, clarified, and agreed upon the key thoughts or contents captured in the codes. They labeled the codes, each reflecting several key thoughts, as the next step. CM and CL sorted the codes into sub-categories based on how the codes were related and linked. They grouped these sub-categories into categories in an iterative process, which continued until they deemed no further abstraction to be appropriate. Subsequently, the co-author MH, who had not previously been involved in the analysis process, critically reviewed the categorization and underlying quotations using selected raw data transcripts to enhance overall trustworthiness [cf. 37]. We utilized member checks, giving all participants the opportunity to comment on the credibility of the presentation of the findings. After critical reading and input from the remaining co-authors, the team revised and finalized the findings.

### Ethical considerations

According to current Swedish legislation, formal ethical approval was not applicable because we did not collect sensitive data. We followed the principles of the Declaration of Helsinki for research involving humans, to the extent relevant for the present study. All participants were given verbal and written information about the study, which included information such as informed consent, confidentiality, the right to not be harmed or identified, and the possibility to withdraw at any time [cf. 38]. All participants gave their verbal and written consent to participate. Interviewing participants about their careers may evoke positive and negative feelings concerning their successes and setbacks. We were therefore vigilant to the participants’ narrations and gave them time to choose what they wanted to share during the interviews [cf. 39]. As part of our ethical considerations, we gave participants the opportunity to read the findings to ensure that confidentiality was preserved. Following the General Data Protection Regulation 2016/679 [40], we protected all data relating to participants with established and accepted principles for data security, and treated it with confidentiality. Only co-workers in the research team had access to the information.

## Findings

We identified three contextual categories: *Well prepared, but still learning and in need of career support and personal guidance*; *Uncertain, while striving toward a balanced and secure working life situation* and *Confident and open-minded, though remaining patient when collaborating and networking*. The participants experienced increased freedom and independence while realizing that they were still in need of support. They strived to find a desired career path, but adapted to available opportunities. Navigating via networking and interdisciplinary collaborations was perceived as a crucial challenges in their present situation.

### Well prepared, but still learning and in need of career support and personal guidance

Several of the participants described how they were still at a learning stage, and perceived having opportunities to learn new skills and increase their interdisciplinary experiences to be essential. Being in a dynamic and creative environment with a variety of disciplines was emphasized as important to enhance the interdisciplinary insights they had achieved during their affiliation with the graduate school. Participating in courses based on interdisciplinary perspectives on ageing and health had made them view their research subject through a different lens and reflect from wider perspectives. They perceived themselves as open-minded rather than attached to a certain viewpoint or method, making them feel well prepared for an early career within interdisciplinary research, despite still being unexperienced.

‘We had a workshop about multidisciplinary, interdisciplinary, and transdisciplinary work, research. And that was quite useful and then I got more of a sense of what [laughter] […]And I felt more confident that I was interdisciplinary, I was not multidisciplinary, not transdisciplinary. I was in between. So I felt that I learned… And in that sense, SWEAH was really good. And also because it’s a group where you have people from different areas doing research… I mean you have this common thing with ageing, researching ageing, but people come from different disciplines. So that also gave me a better sense of what interdisciplinary research means and how we can have this common base and still do so different research.’ (Participant 3)

The transition from doctoral student to early career researcher was perceived as happening directly after the graduation. Most of them experienced an increased sense of freedom compared to during their time as a doctoral student, which implied opportunities to make independent decisions and prioritize engagements and networking. Some stated that this could be a struggle, not knowing whether their choices were beneficial for their careers. Participants emphasized that finding their role and understanding what was expected of them were challenges experienced in their present situation as early career researchers. Some uncertainties concerning what to expect from others, how they themselves were perceived as researchers, and whether they were prioritizing the right commitments were described.

‘…this whole first year after the defense has been a bit like “Wow, who am I? What should I find? What’s my thing here?”, and which way do I want to go. And then you are afraid of not having a job and you jump on a little of everything in some way. But I think now… It’s beginning to feel more like I’m starting to find my way back to my common thread […]. So, parts of it have been there all the time and I have struggled to also find my way back to my core and try to find it.’ (Participant 5)

Even though participants could feel confused in new situations, they did feel ready to take on new tasks and approach new research constellations. However, participants also experienced increased overall confidence and independence in their present working situation. They felt proud and emphasized a new sense of responsibility as an early career researcher. Pushing themselves outside their comfort zones was expressed as vital, and they felt they really had to take on the new role as early career researchers in new situations.

‘I mean, now I’m a postdoc. […]And definitely I am more independent, compared to when I was a PhD student. I’m more independent, I’m also more sure of the content, my way of working, the theories behind… let’s say, ageing in general. So that has changed. But I mean, of course, from being a student to being a postdoc has definitely been an evolution.’ (Participant 15)

Several participants experienced an intensified and more hectic work situation after graduation, with increased responsibilities and tasks to manage. They emphasized the need to adapt their mindsets and adjust to new and diverse contexts and situations. Most of them highlighted that one of the benefits of their affiliation with the interdisciplinary graduate school was encountering a variety of research areas and disciplines, implying the need to explain their own research, methods, and theories to others. Hence, they had been trained to define their research, and what interdisciplinarity meant in their thesis projects. They now realized that this contributed to them being open-minded, not hesitating to approach new areas and perspectives in their present situation.

‘Yes, but maybe this is how I think I probably, through SWEAH… that you defined it more for yourself. I don’t think I thought much about it before I jumped on my PhD project and when I planned it and so on. That it was really interdisciplinary. I think it dawned on me more when I applied to SWEAH and was involved there.’ (Participant 1)

Even though participants perceived themselves as well prepared, they still needed support and guidance, in their day-to-day work and in relation to applying new methods or adjusting to new research contexts.

‘Even if you don’t become a postdoc. In such a position, it would have been good, I think, to have some kind of guidance or program that helps you to profile yourself. Because I think the risk is that you start to spread out in too many directions otherwise.’ (Participant 16)

Participants valued having previous supervisors and/or other senior researchers guiding and supporting them to conduct good quality research and providing them with opportunities to feel included, for example being invited to new working groups and projects, or being part of grant applications and meritorious activities, such as teaching assignments. Participants expressed the need to receive support to make strategic choices and to prioritize and plan the most beneficial path for their career. Having guidance to manage other important aspects of life and finding a balance were also mentioned as important.

‘And that’s something that, maybe if I do another postdoc project, I would like to change. Because I feel a bit alone when working on my project. […] yeah, of course I can ask her [project leader/professor] for opinions and for advice, and she’s quite experienced also, and when I’m certain, “Should I do this or that?” But a lot of the day-to-day work, I need to figure out by myself. So that’s one of the challenges, and also not having other people at the same level as me in the project, maybe.’ (Participant 3)

### Uncertain, while striving toward a balanced and secure working life situation

Because several participants lacked permanent employment, the early career transition was related to work-related uncertainties. They felt that they had to optimize their work engagement so that the time they spent made them follow a beneficial career path. Striving for permanent employment was experienced as being in a sort of limbo situation, and participants recognized that they had to follow a certain path to be competitive. Funding running out and not receiving continuation were factors that constantly occupied several of their minds, which led to an uncertain work situation. Participants perceived this as a catch-22 situation, being in the early career phase and being required to obtain research funding, but having too few merits to be competitive and credible in the eyes of funders. By contrast, those who had received research grants experienced freedom to choose their desired path and described a sense of security.

‘I mean, there is some insecurity in the postdoc position. And that makes me a bit anxious sometimes, because of course you need to depend on the next funding that you’re going to get. And you have these two years to finish your project and then get the next funding. So I feel a bit stressed… I’m always: “Oh, now I’m applying for new funding, I haven’t finished the things I promised for the funding I got.” So, this kind of… This I find difficult to balance.’ (Participant 3)

Participants described that after finishing their doctoral education the career path was not clear, and perceived that there were limited alternatives to follow. One recognized strategy for promoting their career was to get involved in different research endeavours, showing ambition and drive for research. Related to this, some of them had participated in postdoctoral courses offered by the graduate school, which had assisted them in writing their CVs and research grant applications. A few had applied for part-time postdoctoral assignments, including “practical mentorship” offered by the management of the graduate school to support their career development. To foster their career, participants had defined checkpoints and tried to plan strategically to attain these. Examples mentioned included taking the opportunity to supervise doctoral students, teaching, applying for and getting research funding, conducting a postdoctoral period, and shifting research environment and co-workers. These career strategies were perceived as ways to find out what they preferred and found most suitable, and to get a sense of whether they wanted to pursue a career within academia or change path, for example, to the industrial sector. In the early career transition, having opportunities to find their passion, as well as being trusted that they were making progress in their work, were perceived as important aspects to feel that they were driving their career in a strategic direction. Participants recognized that once they had decided on which path to follow, they had to dedicate themselves fully to that path to succeed.

‘I think it has been quite difficult to know “Which path do I choose now, where does it lead? What are the consequences of those decisions?” Also, to know what feels right, I think. Because it was… It’s probably the case for most people that you are right after you have defended your dissertation, and you have to recharge very clearly. And recharge for a future that is actually very uncertain in many ways.’ (Participant 5)

For some, the biggest challenge in the early career phase was to find a balance between time and workload when doing what they really liked and having flexible working conditions. The work situation could become limitless and get out of control when participants wanted to do their best to increase their odds for future grants and employments.

‘The constant challenge is to find some kind of balance in your workload. Because it is easy to want too much. And you know that it is important to have merits. And therefore, you hop on every train. And you see every request for a collaboration as an important opportunity for merit. And then it can become a bit much.’ (Participant 8)

The research world was experienced as tough, competitive, and challenging to navigate. Some participants emphasized the necessity to be mentally strong and hard-skinned, and to push forward, and they struggled to cope with this. Even though participants experienced pressure in environments where achievements were counted all the time, they also recognized this as a driving force to build a career involving tasks that they were passionate about.

‘Yes, the biggest challenge is that the research world is a hard, cold world to operate in. That there is tough competition for money. That there is… sometimes a very harsh, unpleasant tone, because there is just so much competition for money. I mean, that the project money I get has basically been taken from someone else or something. I find it very difficult to reconcile myself with the part of this type of work that is research. So for me it’s probably… I think I’m capable of pushing myself if I want to, but I think for me it’s more about mental toughness. Being hard-headed and pushing on, I think.’ (Participant 13)

### Confident and open-minded, though remaining patient when collaborating and networking

Participants perceived that conducting interdisciplinary research required an open mind, time, and patience. Their previous affiliation with the graduate school was experienced as cutting across diverse perspectives of ageing and health, and meeting people in different career phases. This was perceived as helpful for developing the skills and knowledge necessary for conducting interdisciplinary research and navigating through various environments. Opportunities to discuss ageing and health from their field of research with students and senior researchers from other fields had made them feel confident to apply interdisciplinary approaches and comfortable in interdisciplinary discussions. In their present situation as early career researchers, participants tried to build a bridge between disciplines and achieve a mutual understanding, even though there were different perspectives involved. Trusting that they could find common ground and understanding was a lesson learnt during the graduate school affiliation. They recognized that their view had broadened, and that their respect for others’ perspectives, competencies, and ways of working had been strengthened. Being affiliated with an interdisciplinary graduate school had helped them to feel open-minded and not threatened in their own sometimes narrow research areas. Participants described that when they now met and discussed with researchers from other disciplines within ageing and health, they felt acceptance and respect for other orientations of research, and that they were inclusive, taking into account the benefits of a variety of competencies. They felt familiar with other disciplines’ terminology, and were not afraid or hesitant to collaborate with researchers from different disciplines or to discuss different perspectives of a phenomenon.

‘I think you do it [getting the skills to conduct interdisciplinary research] by just putting yourself in a situation where you are forced to collaborate with others… with researchers from other domains or research topics. And SWEAH has been a significant help in that. Because I think that when you start as a doctoral student in SWEAH, you are very happy to look for those who do the same research as you, because that is where you are most interested. You feel comfortable, you understand what you are talking about and so on. But SWEAH forces you to “No, no, no, you must not be with them”.’ (Participant 10)

During their doctoral education, participants had experienced that they had been assisted with getting into contact with the right expertise when needed. As early career researchers, they had to find and make contact themselves, implying that the responsibility and time spent on networking increased. For some, the comprehensive social aspect of developing their network as a researcher was a new insight. A recurring description was that the graduate school affiliation helped them become more open toward other researchers as potential collaborators rather than competitors. As former doctoral students, participants sensed that they were part of a society of future ageing and health researchers, which made them feel as if they should “stick together”. They did not aim for competition, rather for collaboration in an inclusive spirit.

Most participants recognized that one of the benefits of their former graduate school affiliation was the network they had established, and they also felt that their networking skills had increased. Being part of an interdisciplinary graduate school implied that they created opportunities for future collaborations by establishing and expanding their own network, independently from their research group and former supervisors. Moreover, these interactions increased their overall confidence as researchers and their understanding of the value of having other researchers close by. For most of them, contacts with former fellow students remained after graduation and they perceived the thresholds for approaching colleagues and senior researchers engaged in the graduate school as having been lowered. Having this courage was expressed as important to make contacts and start up collaborations. Participants expressed a desire for structured activities for alumni to keep the vital network alive during the demanding and struggling phase as an early career researcher.

‘…you know, as a PhD student sometimes you are a bit shy, you wait for your supervisors to introduce you to other people. While with SWEAH, it was the opposite because we created the network within SWEAH, and then at conferences […], SWEAH always had some networking session and so on. So it was easier to network through the contacts of people in SWEAH as well. So it’s just that we expanded a bit, the network. And then, even at conferences … let’s say more topic specific, where maybe SWEAH was not involved, in a way you had some tools, some skills that you approach and start talking. So it was a snowball effect in that sense. And I honestly think that if I hadn’t been part of SWEAH, it wouldn’t have been the same.’ (Participant 15)

Participants emphasized that there were many challenges related to collaboration. When collaborating with other researchers, expectations and formal and informal rules were perceived as important to be aware of. Participants recognized that transforming networking into concrete and formal collaboration could be challenging. Especially in interdisciplinary environments, where perceptions of research focus could differ, the challenge was to achieve diverse disciplines to complement each other rather than compromising. Overall, collaboration required patience and was perceived as a slow and strenuous process, which was sometimes questioned by the participants. As expressed by one:

‘You try ten different contacts to get maybe one that respond. […]And sometimes it makes you question: “Why are you wasting time doing this?” Sometimes I just talk at home, like, “Okay, why am I doing this if no one wants to do that?” But then you get one positive e-mail, and you just feel happy with that. And it’s a bit up and down like that, yeah. Sometimes it feels as it could go faster if I just did it myself.’ (Participant 3)

Being part of interdisciplinary collaborative projects and environments required time, because working with people representing different backgrounds or traditions could be time-consuming. Participants described the vital aspect of realizing that working in an interdisciplinary manner could be a long process, before coming to an agreement on what to study, and in what way. Even though working in interdisciplinary environments was perceived as beneficial, it could also be related to feeling out of place and different.

‘I think that my project has been very interdisciplinary. That you are a little bit here and a little bit there. But that’s probably also what makes me feel a bit like this… a bit like the odd one out in my group and so on.’ (Participant 1)

Remaining patient, keeping an open mind, both as a researcher and a person, and understanding the value of including other people’s perspectives were described as key features that were encouraged during their graduate school affiliation.

## Discussion

The findings of this study suggest that early career researchers who participated in the study valued the skills they developed during their prior affiliation with an interdisciplinary graduate school focused on ageing and health. Developing an open mindset permeates the findings as a crucial aspect when it comes to career development in interdisciplinary environments. As reflected in our findings, interdisciplinary graduate schools can be important foundations for nurturing and equipping forthcoming generations of early career researchers with essential skills to navigate and succeed in their career transitions. Based on these findings, and reaching back to the theoretical underpinnings, we suggest the new conceptual coinage of “career ecologies”, in which university–society interconnections are embraced pedagogically and institutionally early in the lives and minds of early career researchers.

While our findings do concur with previous research to some extent, little is seen of the disciplinary academic struggles reported by others [e.g., 13,20]. The graduate school alumni in our study felt well prepared for the career transition following graduation, but were still learning and in need of career support and personal guidance. The former graduate school affiliation implied having established a network within the interdisciplinary ageing and health research field, as well as having the skills and courage to network and make new research contacts. Thus, skills fostered during the graduate school affiliation prepared the participants for work in interdisciplinary research environments.

In contrast to the findings of Balaban [24], where doctoral students were perceived as insufficiently equipped, in terms of their readiness both for professional roles and for their transition to postgraduate life, our study shows that early career researchers who had been affiliated with an interdisciplinary graduate school felt well prepared for their role and responsibilities. Still, our findings imply the need to continue to have opportunities to learn new skills and build on interdisciplinary experiences from doctoral education. That is, even though early career researchers may feel well equipped and prepared for interdisciplinary research collaborations and environments during their doctoral education, this does not seem to be sufficient. To be able to really effectuate the skills developed, they need to be practiced and adapted to the reality early career researchers face after graduation. For example, annual meetings, early career workshops, research visits to other institutions, and inclusive, diverse, and international collaborations have been described as effective learning mechanisms for interdisciplinary research in the early career phase [41,42].

Interdisciplinary researchers move between and across disciplines, which may raise uncertainties related to moving outside the safe space of known areas [43]. Our findings show that interdisciplinary career transitions for early career researchers involve navigating diverse skills and professional networks, which requires adaptability and strategic planning to establish a coherent and sustainable career path. As seen in our study and as supported by Dupont et al. [44] and Lyall and Meagher [45], this entails a need for structure and guidance in the early career stage, especially when there are requirements and expectations to navigate the space between disciplines [cf. 46]. These findings offer valuable insights for interdisciplinary academic mentoring and leadership practices, potentially shaping career counseling and personal development strategies within educational institutions.

Our findings are very much in line with aspects that are important for career development as described in the Vitae Researcher Development Framework [8], such as communication, collaboration, self-confidence, and networking. Still, the findings also implied struggles with uncertainty and how to strategically prioritize and balance working life. Being an early career researcher has been described as an emotional journey that requires commitment and resilience [1,25,47]. Career resilience, especially for early career researchers in interdisciplinary fields, is crucial for managing the uncertainties and pressures associated with the academic transition from student to researcher. As interdisciplinary career paths often lack the structured support typically found in traditional disciplines, researchers have to rely on self-motivation, adaptability, and strategic networking [30]. Being resilient is crucial during the career transition, as it enables individuals to maintain self-belief, persist through challenges, work hard, and stay motivated to seize opportunities for vital meritorious career steps [48]. Related to our findings, interdisciplinary graduate schools can play a crucial role in fostering resilience by preparing students to navigate these challenges. Building resilience in interdisciplinary contexts not only helps early career researchers navigate through emotional challenges and career uncertainties, but also helps them make thoughtful, strategic decisions that align with both personal and professional growth trajectories [30]. This support empowers early career researchers to manage career transitions and development with greater confidence and adaptability, ultimately contributing to healthier and more successful career paths.

Our study shows that participants felt part of a community due to their graduate school affiliation, which inspired them to develop their network in a confident and open-minded manner. Gardner et al. [21] have shown that support from peers is essential for doctoral students to have opportunities to build a community and navigate challenges concerning interdisciplinary research training. Our study contributes to the understanding of how structured interdisciplinary graduate schools shape the transition from graduate student to early career researcher, and provides insights into the role of such schools in fostering a strong sense of community, interdisciplinary confidence, and professional identity. Related to the findings of the present study, one factor supporting this may be the shared foundation in research on ageing and health. The sense of belonging and common purpose cultivated within the graduate school contributed to building a community among the doctoral students that extended beyond the doctoral student period. This lasting sense of community with other alumni and mentors may serve as an important resource for navigating the early career researcher stage [cf. 30]. The support of a larger community has the potential to strengthen individuals’ adaptability and resilience. Strengthening these skills is important for career preparation, and to face challenges confidently. Moreover, being part of a connected community fosters shared growth, mentorship, and a collective resilience that enhances personal development [30].

Related to our findings and turning to the literature [e.g., 11,14,15,28,31], interdisciplinary identity formation is shaped by continuous negotiation between disciplinary boundaries, professional networks, personal experiences, and adapting to evolving career paths. What an interdisciplinary graduate school adds might be a community with shared values, collaborative exchanges, mutual support, and the exchange of diverse perspectives fostering personal and professional development, nurturing interdisciplinary identity formation. Thus, the potential to feel a sense of belonging to a like-minded community may support individual growth and building on these shared interests together [cf. 46]. Hence, this sense of belonging cultivated within the graduate school may foster resilience and a shared commitment to interdisciplinary problem solving – outcomes less often addressed in previous research.

The findings and conceptual development contribute to the literature by moving the understanding of career transitions away from a linear and step-by-step way of thinking, where the career dimension only begins to play a part late in the academic learning trajectory. With the concept of career ecologies, we argue that academic careers should be understood in an entangled or interlinked way, where universities and their various curricula are intertwined and nested within wider societal contexts from the very beginning. Rather than adding further pressure to an already complex and demanding academic journey, our point is that the consciousness of career ecologies has the potential to reduce complexity and create stronger cohesion and focus in the academic formation process. The concept of career ecologies should ideally encourage, empower, and energize doctoral students and early career researchers, urging them to activate and make use of their societal voice, influence, and value right from the early formative doctoral years.

## Methodological strengths and limitations

Using the comprehensive Researcher Development Framework [8] to cover a diversity of domains of academic skills and developments, the explorative study design made it possible to ask graduate school alumni complex and integrative questions about their career transition after graduation. Content analysis [36] provided opportunities to gain a comprehensive understanding of the topics at target.

Data were collected and analyzed by a former study coordinator (CL) and an alumna (CM) from the graduate school that constituted the study context. Their prior beliefs and familiarity with the learning environment could be seen as both a strength and a weakness. On the one hand, they were well-versed in the context of the graduate school, but on the other hand they may have held biases. For example, their pre-understanding might have influenced the data by eliciting responses that were less critical and inclined toward positivity. Throughout the study, CM and CL maintained a reflexive approach to critically examine how their own social position, experiences, and assumptions may have influenced the co-construction of knowledge with participants [cf. 36]. Trustworthiness and credibility were strengthened through the involvement of co-author MH, who applied a critical review and validity check of findings, as well as through member checking with the participants. Maintaining reflexivity within the entire team of authors, the involvement of senior researchers representing different types of expertise as co-authors strengthened the trustworthiness and credibility even further.

While the findings suggest that the graduate school offered opportunities for interdisciplinary engagement among the participants, it is possible that some of the distinctive characteristics attributed to their experiences were, in fact, shaped by the specific context of their professional environments after graduation. Additional research might consider whether or how different work environments shape interdisciplinary researchers early in their career.

The interviews were conducted during the COVID-19 pandemic, which may have impacted on participants’ experiences of their early research career and transition, for example due to restrictions related to travelling and the possibilities to start research projects with new collaborators.

## Conclusions

The present study shows that being affiliated with an interdisciplinary graduate school has the potential to foster a future generation of scholars and appears to create a supportive environment that endures even after their doctoral studies. Introducing a new conceptual coinage of “career ecologies”, we suggest that academic careers should be understood in an entangled or interlinked way from the very beginning of the academic journey. This implies that doctoral students and early career researchers should be encouraged, empowered, energized, and invited to activate and make use of their societal voice, influence, and value right from the early formative doctoral years. The findings indicate that structured interdisciplinary programs are critical platforms for cultivating the skills and identities necessary for impactful research and collaboration in a complex, interconnected world. Moreover, this study emphasizes the need for continued support considering the diversity of challenges met by early career researchers. The need for support after graduation is considerable, and calls for explicit and strategic attention.

## Supporting information

S1 Appendix Interview guide. The full interview guide utilized in the study.
(DOCX)
