# Supplementary material for: Well prepared yet uncertain: Experiences of the early career transition after affiliation with an interdisciplinary graduate school
Source: PLoS One. 2025 Apr 29;20(4):e0321039. doi: 10.1371/journal.pone.0321039 (PMC12040177; doi:10.1371/journal.pone.0321039)
Supplement: S1 Appendix — The full interview guide utilized in the study. (DOCX) [file pone.0321039.s001.docx]

**S1 Appendix. Interview guide.** The full interview guide utilized in the study.

Can you want to start by telling me how you feel now, x number of years / months after your dissertation? What is your work situation look today? What is your research environment like today, compared to as a doctoral student?

- How has your work situation changed since your dissertation?
- What is your research context like now after your dissertation, compared to before?
- If you had to change something about your current situation, what would you change?

**Engagement, influence and impact**

- Can you tell me what it is like to navigate the postdoc world? To find your own position? Are there accessible routes to get where you want to be? Is there anything that has surprised you? Have you encountered anything you did not expect?
- Can you tell me about how you collaborate with colleagues or researchers in your own area or other areas?
- How does collaboration take place? (Joint projects, applications, publications?)
- How do you experience/value this collaboration? Opportunities? Challenges? Why? How does collaboration with different areas contribute to your research?
- What significance has the network that you built up during your SWEAH period had for your current situation? Have you had contact? Active collaboration? Are you planning on it? Why?
- What do you think about networking today? Has your view of networking changed? If so, how?
- How do you view the competence required to conduct interdisciplinary research? How do you get it? Do you feel that you have the right skills/qualifications/experience for it? In what way has SWEAH played a role in developing this?

**Research governance, organization and personal effectiveness**

- What do you think you will be doing in five years? What does it mean for you to build a career? What do you think about that? What career success do you feel you have had? Publications? Own research grants? Change university/research group? Supervising doctoral students? Leading projects?
- If you could control your career development, what would your goals ba and in which direction would you like to go? Own new research ideas/line? Self-confidence? Security? Motivation?
- What drives you in your career? What do you want to achieve or accomplish? (Reflect on goals and values). How can you qualify for new assignments/management?
- If you were to create your own research group/line, how would you do this? What is important for good quality in research to be competitive? What type of collaboration/project, methods, design? When is the right time for your own research line/group?
- What are the challenges involved in getting where you want to be (to reach your goals)? Organizational structural practice within the university and faculty, personal/career goals, values, research finances, labor market.

**Knowledge and intellectual abilities**

- Can you tell me about how you experienced your time as a SWEAH-affiliated doctoral student in retrospect? What specifically was most important? (Doctoral student days, courses, other SWEAH events – was it, for example, taking the course or entering a new environment and presenting your research to others than at your home university?)
- Looking back, how – and in what way – do you feel that your time at SWEAH (the skills you developed) has affected you and what you do today? Why do you think that is? Can you give any examples? What do you think about your continued competence development regarding breadth in ageing and health?
- Since your thesis defense – that is, as a postdoc at SWEAH – has SWEAH contributed to your postdoc experience in any way? How? (Postdoc assignments? Courses? Other?)
- Would you have done anything different if you participated in SWEAH again to prepare for your postdoc period? Why? How?

Finally, is there anything else you have thought of that you would like to tell us about?
